# Gene Therapy: A Promising Approach for Neuroprotection in Parkinson’s Disease?

**DOI:** 10.3389/fnana.2016.00123

**Published:** 2016-12-20

**Authors:** Pamela Valdés, Bernard L. Schneider

**Affiliations:** Brain Mind Institute, Ecole Polytechnique Fédérale de Lausanne (EPFL)Lausanne, Switzerland

**Keywords:** Parkinson’s disease, gene therapy, neuroprotection, risk factors, mitochondria, autophagy, neurotrophic factors

## Abstract

With the development of effective systems for gene delivery to the central nervous system (CNS), gene therapy has become a therapeutic option for the treatment of Parkinson’s disease (PD). Gene therapies that are the most advanced in the clinic have been designed to more effectively compensate for the lack of dopamine signaling in the basal ganglia and rescue the cardinal motor symptoms of PD. However, it remains essential to devise novel therapies to prevent neurodegeneration and disease progression. Since gene therapy has been initially proposed for the delivery of neurotrophins to support the survival and function of dopaminergic neurons, our understanding of PD etiology has changed dramatically. Genes implicated in familial forms of the disease and genetic risk factors associated with sporadic PD have been identified. The spreading of the α-synuclein pathology, as well as perturbations of the lysosomal and mitochondrial activities, appear to play critical roles in the pathogenesis. These findings provide novel targets for gene therapy against PD, but at the same time underline the complexity of this chronic disease. Here we review and discuss the successes and limitations of gene therapy approaches, which have been proposed to provide neuroprotection in PD.

## Introduction

Parkinson’s disease (PD) is a progressive neurodegenerative disease which mainly affects the central nervous system (CNS) and in particular the basal ganglia. The most salient histopathological feature of PD is the widespread deposition of the misfolded α-synuclein protein in the brain, and already at early stages of the disease, in neurons innervating peripheral organs, such as the gut and the heart (Klingelhoefer and Reichmann, 2015). The most studied and best understood degenerative process caused by PD is the loss of dopaminergic neurons localized in the *substantia nigra pars compacta*, and projecting to the *striatum*. Indeed, nigral neuron loss leads to deficits in the initiation and control of voluntary movements, which represent the cardinal symptoms of PD.

**Figure 1:**
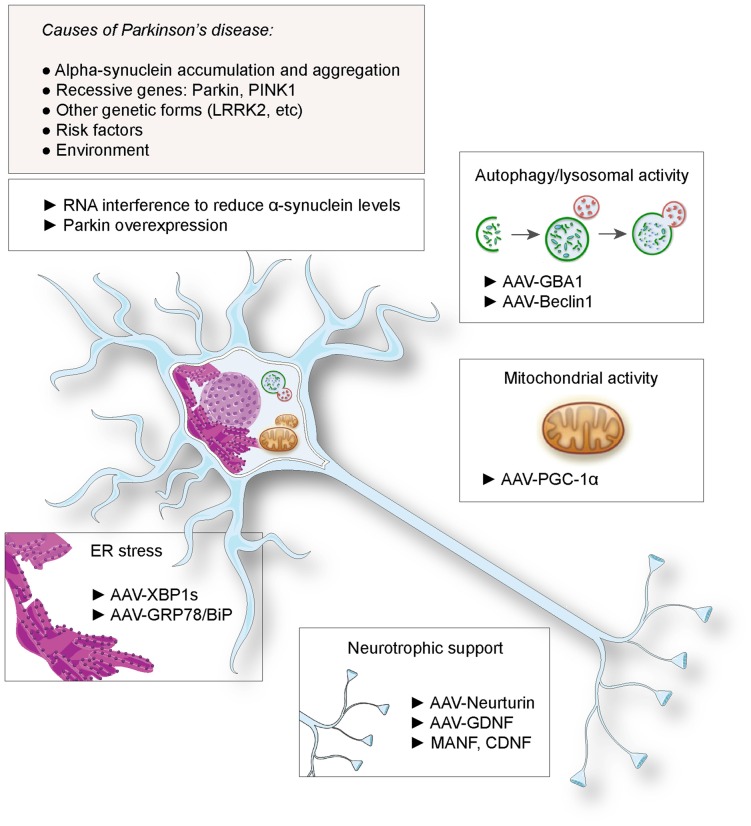
**Proposed gene therapies for neuroprotection against Parkinson’s disease (PD).** Based on the identified causes and risk factors for PD, various strategies have been envisaged, including RNA interference against α-synuclein and Parkin overexpression. Gene transfer has been proposed to deliver neurotrophic factors to rescue dopaminergic function, factors to suppress endoplasmic reticulum (ER) stress, enhance lysosomal and mitochondrial activities.

Gene therapy has been considered as a promising therapeutic approach for PD. This notion has been spurred by the possibility to genetically modify neuronal populations of the basal ganglia, to compensate for the lack of dopamine release and rescue the activity of the circuit controlling movement initiation. Indeed, gene delivery systems, mainly based on the viral vector technologies, have demonstrated their efficacy for gene transfer to nuclei critically involved in the disease, such as the *substantia nigra*, the subthalamic nucleus or the *striatum*. The most advanced therapeutic approaches based on gene delivery aim at alleviating the motor symptoms or prevent the occurrence of dyskinesia, by improving the efficacy of dopamine replacement (Coune et al., 2012). Notably, Prosavin has recently reached a phase I/II clinical trial, demonstrating efficacy in restoring dopamine levels in the *striatum*, which may represent an appealing alternative to levodopa administration (Palfi et al., 2014). This therapy is based on an equine lentiviral vector encoding tyrosine hydroxylase, GTP cyclohydrolase and aromatic acid decarboxylase (AADC), the three enzymes needed for dopamine synthesis from L-tyrosine (Azzouz et al., 2002). However, continuous neurodegeneration remains a major issue over time. The loss of neurons in the *substantia nigra* and other brain regions decreases the efficacy of dopamine replacement therapies and ultimately leads to the emergence of non-motor symptoms such as sleep disorders, as well as mood and cognitive dysfunctions. Based on the genetic causes of PD, or risk factors involved in disease etiology, novel approaches have been proposed to protect neurons and slow down the course of the disease (summarized in Figure 1). Although causal genetic mutations account for only 10% of the patients, the familial forms of the disease could represent a first rational target for gene therapy. Furthermore, neuroprotective factors that are not directly involved in the pathogenesis, such as neurotrophic factors, have also been considered. Based on these examples, we discuss the use of gene delivery as a neuroprotective approach in PD.

## Why Considering Gene Therapy for Neuroprotection Against Parkinson’S Disease?

Historically, PD has been a major focus for gene therapy in the CNS. The use of gene delivery as a potential treatment has been mainly driven by the possibility to target the nigrostriatal system, which degenerates in PD and is responsible for the main motor symptoms. The *substantia nigra* contains a population of less than 500,000 dopaminergic neurons per hemisphere in humans, and their cell soma are distributed within a small volume of the ventral midbrain (Rudow et al., 2008). Nigral dopamine neurons develop an extensive axonal arborization in the *striatum*, a key structure in the basal ganglia. It is feasible to locally inject viral vectors to genetically modify these neurons, or glial cells localized in their direct environment. Both Adeno-associated virus (AAV) and lentiviral vectors have proved highly effective to deliver genes either in the *substantia nigra* or in the* striatum* (Lundberg et al., 2008; Löw et al., 2013). Therefore, the administration of neuroprotective genes has appeared as a realistic therapeutic option. At the same time, the development of symptomatic treatments to restore the proper function of the basal ganglia, such as chronic levodopa administration and deep brain stimulation, has made constant progress. The significant effects of these treatments on the motor symptoms of PD raise the bar for gene therapy. Indeed, the risk-to-benefit balance has to be carefully addressed for treatments that are based on permanent gene delivery, a technology which is still considered risky for conditions that are not life-threatening. However, as none of the existing treatments can prevent PD progression, there is still a major unmet therapeutic need.

The past two decades have witnessed major developments in our understanding of PD etiology. In particular, a number of genes have been found to have a causal role in familial forms of PD, including α-synuclein, Leucine-rich repeat kinase 2 (LRRK2), parkin and PTEN-induced putative kinase 1 (PINK1) (for review see Hernandez et al., 2016). Other genes, such as β-glucocerebrosidase (GBA1), are considered as risk factors for the disease and may also offer opportunities for gene therapy. Altogether, although PD does not appear to be linked to a single pathway, these genetic factors point to changes in mitochondrial and lysosomal activities as a key axis in the pathogenesis. However, uncertainty prevails regarding how to address these pathogenic mechanisms using gene therapy.

### What Are the Genetic Causes of Parkinson’s Disease That Can Be Targeted by Gene Therapy?

The overabundance of α-synuclein, a small presynaptic protein, is considered a major pathogenic mechanism in both genetic and sporadic forms of PD. The rare familial forms linked to mutations and multiplications of the α-synuclein gene, as well as the finding that α-synuclein is the main constituent of Lewy bodies, support this notion. Overall, conditions leading to the misfolding of this protein may contribute to the disease. The level of α-synuclein is therefore considered to be a critical factor. Gene therapy has been proposed for RNA interference against α-synuclein, assuming it would be possible to substantially decrease the level of the protein without inducing major side effects (Khodr et al., 2011, 2014). However, silencing of α-synuclein has led to nigral degeneration in rats and primates, a robust effect which is at odds with the minor phenotypes observed in α-synuclein-null mouse strains (Gorbatyuk et al., 2010; Collier et al., 2016). Furthermore, α-synuclein has been found implicated in vesicular neurotransmitter release (Burré, 2015), suggesting that the level of α-synuclein may have to be carefully adjusted to avoid defects in neurotransmission. Overall, controlling α-synuclein levels in neurons by gene therapy appears as a challenging task, which will require a better understanding of the mechanisms that control the homeostasis of this protein in health and disease.

Genes involved in recessive forms of familial PD may represent easier targets for gene therapy, with the aim to rescue proper gene function. In particular, loss-of-function mutations in the genes encoding Parkin and PINK1 are together responsible for a significant number of recessive PD cases. These two genes are active in the same pathway (Clark et al., 2006; Park et al., 2006). Although the overall role of this pathway in selectively vulnerable populations of neurons remains unclear, there is increasing evidence for its involvement in the control of mitochondrial turnover, by inducing mitophagy via the ubiquitination of mitochondrial proteins (Narendra et al., 2008). As loss-of-function mutations can be potentially rescued by overexpressing the functional protein, this may represent a possible candidate application for gene therapy in PD. However, patients who carry Parkin mutations have an atypical form of Parkinsonism characterized by early onset and slow disease progression (Lohmann et al., 2003). Lewy bodies positively stained for α-synuclein are in most cases not observed in the *substantia nigra* (Mori et al., 1998). In these patients, the motor symptoms can be efficiently corrected by levodopa administration. The efficacy of the symptomatic treatments opposes the use of gene therapy for the rescue of Parkin activity as a possible alternative treatment. Furthermore, it remains unclear if chronically enhancing the activity of the PINK1-Parkin pathway may represent a therapeutic option for sporadic PD cases, as suggested by the neuprotective effects of Parkin overexpression on neurons exposed to α-synuclein toxicity (Lo Bianco et al., 2004b; Yasuda et al., 2007). Indeed, the long-term chronic overexpression of Parkin may even cause adverse effects such as the loss of neurons positive for dopaminergic markers in the nigrostriatal system, which raises the need for careful vector dosing (Van Rompuy et al., 2014).

Although the therapeutic use of gene delivery appears challenging in the context of PD, the expanding list of genes implicated in PD may reveal novel options for gene therapy. In addition, the use of novel targeted gene editing technologies, such as the bacterial Clustered regularly interspaced short palindromic repeats (CRISPR)-associated protein-9 nuclease (Cas9), provides novel modalities for more precise intervention on the genetic defects that underlie the disease. It is therefore plausible that gene therapy may ultimately find application for neuroprotection in the familial forms of the disease.

## Identifying Key Downstream Targets

### Autophagy

Although pathogenic pathways implicated in PD are intensively investigated, the possible mechanisms that may represent common denominators between genetic and idiopathic PD have remained rather elusive. Nevertheless, some risk factors have been identified that appear to be critical mediators of the lysosomal and mitochondrial activities. The *GBA1* gene encodes the lysosomal enzyme β-glucocerebrosidase (GCase), which is involved in the conversion of glucosylceramide into glucose and ceramide. Whereas the complete loss of GCase activity leads to Gaucher disease, heterozygous carriers of *GBA1* mutations have increased risk of developing PD (Neumann et al., 2009). During aging and in PD, a progressive decrease of GCase activity is observed (Rocha et al., 2015a). Furthermore, reduced GCase activity leads to enhanced levels of α-synuclein, which in turn further affect GCase (Mazzulli et al., 2011). This vicious circle leads to impaired lysosomal function. A possible therapeutic approach is to rescue GCase activity (Mazzulli et al., 2016). As it is difficult to administer the recombinant enzyme to the CNS, an alternative approach is to deliver *GBA1* by gene therapy. In the rat *substantia nigra* overexpressing human A53T α-synuclein, co-injection of an AAV vector encoding GCase has neuroprotective effects on dopaminergic neurons (Rocha et al., 2015b). Furthermore, the same vector injected in transgenic mice overexpressing wild-type human α-synuclein led to a reduction of the high molecular weight α-synuclein species, and decreased the insoluble α-synuclein aggregates in the *substantia nigra* and *striatum*.

Alternatively, it is also possible to enhance autophagy by overexpressing critical factors controlling lysosomal activity. In particular, the transcription factor TFEB, which regulates genes involved in the “Coordinated Lysosomal Expression and Regulation” (CLEAR) network, has been found clustered in the cytosol of diseased dopaminergic neurons (Decressac and Björklund, 2013). Markers of autophagic activity were found to be dysregulated in neurons overexpressing α-synuclein. Based on these observations, Decressac et al. (2013) found that AAV-mediated expression of either TFEB or Beclin-1, another key regulator of the autophagy-lysosome pathway, had clear neuroprotective effects on rat nigral dopaminergic neurons overexpressing human α-synuclein. Notably, the injection of these viral vectors induced local protection of both neuronal cell bodies and their striatal projections, and improved dopamine neurotransmission. Considering that the activities of GCase and transcription factor EB (TFEB) could be interdependent (Sardiello et al., 2009; Awad et al., 2015), these studies highlight the possibility of locally enhancing the autophagy-lysosome activity by gene therapy, to provide neuroprotective effects against the accumulation of α-synuclein. Furthermore, restoring lysosomal activity may also reduce intercellular transmission of the α-synuclein pathology, which may affect disease progression (Kim et al., 2016). However, before envisaging gene therapy to increase autophagy-lysosome activity, it will be critical to explore the long-term effects, as deregulated autophagy can lead to neuronal cell death.

### Mitochondrial Activity

PD has been notoriously associated with defects in the mitochondrial function. A notion further supported by the effects of mitochondrial toxins, such as rotenone and 1-methyl-4-phenyl-1,2,3,6-tetrahydropyridine (MPTP), which lead to syndromes characterized by the selective degeneration of dopaminergic neurons in the *substantia nigra*. More recently, factors implicated in familial forms of PD, such as Parkin/PINK1 and α-synuclein, have also been found to directly affect mitochondria. In line with the primary role of mitochondria in PD, a meta-analysis of gene expression changes found in the *substantia nigra* identified a set of genes implicated in bioenergetics to be consistently downregulated in PD (Zheng et al., 2010). In particular, several of these genes share Peroxisome proliferator-activated receptor gamma coactivator 1-alpha (PGC-1α), a master regulator of mitochondrial biogenesis, as a common factor regulating their transcription. Therefore, PGC-1α has been proposed as a therapeutic target in PD, which is further supported by the direct interaction between PGC-1α activity and the α-synuclein pathology and by the finding that Parkin controls PGC-1α expression via the proteasomal degradation of PARIS, a transcriptional repressor of PGC-1α (Shin et al., 2011). AAV-mediated gene therapy to provide neuroprotection by increasing PGC-1α expression has been addressed in rodent models of PD. The neuroprotective effects were tested in neurons either overexpressing human α-synuclein, or exposed to the MPTP neurotoxin (St-Pierre et al., 2006). Although significant protection was achieved in acute models of neurotoxicity, these experiments also revealed a clear down-regulation of dopaminergic markers following chronic expression of PGC-1α at supraphysiologic levels (Ciron et al., 2012). This effect was linked to a down-regulation of Pitx3, a factor controlling the expression of important genes in the differentiation of dopamine neurons (Clark et al., 2012). When PGC-1α was expressed at high levels for several months in the rat *substantia nigra*, selective degeneration of nigral dopaminergic occurred, possibly caused by the accumulation of defective mitochondria (Ciron et al., 2012). Remarkably, PGC-1α null mice are more susceptible to PD-related stress, in particular to the toxic effects of human α-synuclein overexpression (Ciron et al., 2015). In this context, which perhaps mimics more closely the pathologic conditions of PD, AAV-mediated expression of PGC-1α had protective effects against α-synuclein-induced neurodegeneration (Ciron et al., 2015). These results underline the need to maintain the level of PGC-1α activity within a therapeutic window, which may be difficult to achieve over the long term via gene therapy.

### ER Stress

Among the pathogenic mechanisms that affect neurons in PD, stress of the endoplasmic reticulum (ER) and the subsequent unfolded protein response (UPR) may play an important role. ER stress is broadly observed in several models of the disease (Mercado et al., 2013), and markers of ER stress and activation of the UPR have also been detected in *post-mortem* tissue and iPSC-derived neurons obtained from PD patients (Chung et al., 2013). ER stress is therefore emerging as a potential pathogenic mechanism, which raises the possibility of targeting the UPR as a potential therapeutic approach. In mammals, upon stress caused by the presence of misfolded proteins in the ER lumen, the UPR is triggered by the activation of three transmembrane sensor proteins: IRE1α, ATF6 and PERK. Once activated, these sensor proteins induce the activation of three transcription factors, XBP1s, ATF6f and ATF4, which translocate to the nucleus to regulate the expression of genes involved in protein folding and quality control, protein secretion, autophagy, protein degradation and ultimately apoptosis (Walter and Ron, 2011). Under acute ER stress conditions, the UPR mediates cellular adaptation to restore ER homeostasis. However, when the stress becomes chronic, the same pathway can trigger apoptosis to eliminate the damaged cells.

Several approaches have been considered to decrease the levels of ER stress and control the UPR by gene therapy. One strategy could be to directly target the accumulation of misfolded proteins in the lumen of the ER of dopaminergic neurons to avoid the activation of pathogenic downstream mechanisms. Accumulation of α-synuclein in experimental rodent models leads to ER stress and activation of the UPR. In this context, Gorbatyuk et al. (2012) delivered an AAV vector into the SNpc to induce overexpression of GRP78/BiP, an ER-resident chaperone modulating ER stress. GRP78/BiP was found to promote the survival of nigral dopaminergic neurons in rats overexpressing α-synuclein, with a decrease of ER stress and apoptotic markers (Salganik et al., 2015). Gene therapy was able to protect neurons, rescue dopamine levels in the *striatum*, and significantly correct the motor impairments observed in this animal model.

Alternatively, it is also possible to selectively modulate one arm of the UPR by gene therapy. Target selection in this complex pathway is not a simple task, and the effects of the factors critically involved in UPR are poorly predictable. Silva and collaborators showed that in mice deficient for C/EBP homologous protein (CHOP), a transcription factor implicated in apoptosis caused by ER stress and activated by the PERK pathway, dopaminergic neurons are protected against MPTP (Silva et al., 2005). However, as this work was based on CHOP null mice, it is also possible that compensatory mechanisms have contributed to the observed neuroprotection. Although gene therapy approaches to block CHOP activity have not been tested yet, one study was based on the AAV-mediated overexpression of ATF4, a transcription factor activated by PERK and upstream of CHOP (Gully et al., 2016). ATF4 expression induced the loss of dopaminergic nigral neurons in a rat model of PD, suggesting that this arm of the UPR pathway could indeed play a critical role in the degeneration of these neurons.

Decreasing the overload of misfolded proteins at the ER could also be achieved through the activation of the IRE1α pathway. Two studies attempted to genetically manipulate this UPR arm as a potential therapy for PD. XBP-1s is a transcription factor activated by IRE1α and that controls a subset of genes involved in protein folding, ER/Golgi biogenesis and ER-associated degradation of proteins. The delivery of an AAV vector encoding XBP-1s into the *substantia nigra* increases the survival of dopaminergic neurons in two different neurotoxin-based models of the disease (Sado et al., 2009; Valdés et al., 2014). Although the exact protective mechanism remains elusive, it is likely that the activation of this UPR pathway can alleviate the ER stress triggered by the neurotoxins. Moreover, as the silencing of XBP-1s in adult mice was shown to rapidly trigger the death of nigral dopaminergic neurons, this factor seems to play a critical role in the maintenance and survival of these neurons even in physiological conditions (Valdés et al., 2014). Gene therapy based on the delivery of XBP-1s is a promising approach for PD. However, the multifaceted roles of this factor have to be carefully considered before envisaging chronic activation of this pathway. Notably, a recent study has shown a role of XBP-1s in memory formation, indicating that potent effects are to be expected in various neuronal systems (Martínez et al., 2016).

## Neuroprotection with Genes Encoding Neurotrophic Factors

Gene delivery has been used to express neurotrophins, as these proteins typically poorly penetrate the brain parenchyma. The delivery of neurotrophic factors by gene therapy has proved very effective for neurorestoration of dopaminergic neurons in the context of PD. Local delivery of neurotrophins, such as glial cell derived neurotrophic factor (GDNF) and neurturin, leads to enhanced dopamine synthesis, axonal sprouting and the generation of novel synaptic connections, which translates into improved motor function. The restorative effect of neurotrophins is particularly evident following acute and selective lesions of the nigrostriatal dopaminergic system with selective toxicants, such as 6-hydroxydopamine (6-OHDA) and MPTP (Kordower et al., 2000). However, it remains unclear whether neurotrophic factors can provide effective protection in PD. Notably, the effects of GDNF was found to be dramatically reduced in presence of the α-synuclein pathology (Lo Bianco et al., 2004a; Decressac et al., 2011). Impaired GDNF signaling is caused by the decreased expression of the transcription factor Nurr1 in dopaminergic neurons overexpressing human α-synuclein, which leads to lower expression of the GDNF receptor Ret (Decressac et al., 2012). A gene therapy approach combining Nurr1 and Foxa2 has recently been tested in MPTP-treated mice (Oh et al., 2015). Neuroprotective effects were obtained, lasting for up to 1 year after AAV vector injection. However, the efficacy of this approach remains to be tested in genetic models of PD.

AAV-based gene therapy has been used in PD patients to deliver neurturin, a neurotrophic factor which is part of the GDNF family, with a similar mode of action. AAV2-neurturin (CERE-120) was tested in one open-label phase I trial followed by a phase II trial, using bilateral intraputaminal delivery of the vector in moderately advanced PD patients (Marks et al., 2008, 2010). The results did not demonstrate any significant benefit in the UPDRS score compared to the sham-operated group. As there was little expression of neurturin in the *substantia nigra*, a follow-up trial assessed patients injected with the vector in both the putamen and the *substantia nigra* (Warren Olanow et al., 2015). However, there was no significant effect of AAV2-neurturin compared to sham surgery. A trial is currently ongoing to test the effect of a similar AAV2-GDNF vector.

Overall, it remains unclear whether the delivery of neurotrophic factors by gene therapy has the potential to slow down disease progression in PD. The results of the initial trials have been disappointing, and it is unclear if neurotrophic factors of the GDNF family can exert protective effects in the context of the α-synuclein pathology. However, novel neurotrophins with different modes of actions have been found, such as mesencephalic astrocyte derived neurotrophic factor (MANF) and cerebral dopamine neurotrophic factor (CDNF; Petrova et al., 2003; Lindholm et al., 2007). These factors have potent trophic effects on dopaminergic neurons. In addition, they reside in the ER, where they interact with GRP78/BiP and control ER stress (Lindahl et al., 2016). It will be critical to determine the effects of these factors in the context of the PD pathology, and if they can be safely delivered by gene therapy (Bäck et al., 2013).

## Conclusions

The use of gene therapy for neuroprotection in PD has to be continuously reassessed in the light of the recent findings regarding disease etiology. While genes implicated in the familial forms of the disease represent the most rational targets for gene therapy, it has been difficult to establish proof-of-concept for rescue approaches. The disease appears to perturb lysosomal and mitochondrial activities that are essential to the long-term maintenance of neuronal homeostasis. Neuroprotective approaches to support these pathways often cause unexpected effects, in particular when genetic modifications lead to long-lasting changes in the expression of key factors. In addition, it is now considered that misfolded and aggregated α-synuclein deposits that represent a hallmark of PD pathology, may propagate through neuronal systems in the peripheral and CNS, via mechanisms that remain unclear. The pathology appears no more restricted to the nigrostriatal system, and the motor symptoms linked to the selective vulnerability of the dopamine neurons, and which have been the main target for gene therapy so far, are likely to be the tip of the iceberg. Hence, PD should be considered as a complex syndrome that will likely require combined therapeutic interventions for effective treatment. Novel gene delivery tools show unprecedented efficacy in the CNS, and more precise techniques are currently being developed for genetic correction. By supporting the survival of neurons that are chronically exposed to the disease, it is likely that gene therapy will become part of the arsenal needed for neuroprotection.

## Author Contributions

PV and BLS wrote and edited the present manuscript and designed the figure.

## Funding

This work was supported by Swiss National Science Foundation Grant No 31003A_120653 and 31003A_135696.

## Conflict of Interest Statement

The authors declare that the research was conducted in the absence of any commercial or financial relationships that could be construed as a potential conflict of interest.
